# Association of Life’s Crucial 9 with the risk of all-cause and cardiovascular mortality among US patients with stroke

**DOI:** 10.1097/MD.0000000000044690

**Published:** 2025-09-26

**Authors:** Wenbo Zhang, Wanhong Zhang, Henghao Wu, Jiaying Li, Xinsheng Han

**Affiliations:** a Department of Neurology, Kaifeng Central Hospital, Xinxiang Medical University, Kaifeng, China; b Department of Neurosurgery, Kaifeng Central Hospital, Kaifeng, China; c Department of Nursing, Shangluo College, Shangluo, China; d Department of Neurology, Henan Key Laboratory of Neuromuscular Pathology, Kaifeng Central Hospital, Kaifeng, China.

**Keywords:** cardiovascular mortality, LC9, NHANES, stroke

## Abstract

Previous studies have demonstrated an association between the Life’s Crucial 9 (LC9)-based cardiovascular health score and all-cause and cardiovascular mortality in individuals without cardiovascular disease. Higher scores have been associated with reduced all-cause and cardiovascular mortality. However, no study has explored the association between the LC9 and the survival outcomes in stroke patients. This study aimed to analyze the association between the LC9 and all-cause and cardiovascular mortality in the American stroke population. Based on the National Health and Nutrition Examination Survey database from 2007 to 2018, a total of 949 eligible patients were enrolled in our cohort study. The relationship between LC9 and mortality was analyzed using univariate weighted Cox proportional regression analysis models and Kaplan–Meier curves. A subgroup analysis was conducted to ascertain whether there was an interaction between age, sex, education, hypertension, and diabetes. The restricted cubic spline curve was employed to examine the association of LC9 with risk of death. The study cohort comprised 949 patients who had experienced a stroke, of with 313 died from all causes and 89 died from cardiovascular causes. In the Cox regression model, after adjusting for confounding factors, a significant inverse association was observed between the highest third of the patients’ LC9 and all-cause mortality (95% confidence interval 0.35–0.78, *P* = .001) and cardiovascular mortality (95% confidence interval 0.12–0.56, *P* < .001). Furthermore, a negative association was identified between the LC9 and patient mortality rate, indicating that the higher the LC9 score, the lower the mortality rate. The Kaplan–Meier curve showed that the higher LC9 score, the better prognosis for the patient and a reduced mortality rate. No interaction was identified in the subgroup analysis. Dose-response analysis also showed that LC9 was linearly and negatively associated with all-cause mortality and cardiovascular mortality in patients.

## 1. Introduction

Stroke represents a significant global public health concern, encompassing both ischemic and hemorrhagic stroke. It is the second leading cause of mortality and the third leading cause of disability worldwide.^[1,2]^ In 2021, statistics indicated that approximately 7.3 million deaths were attributed to stroke, representing 10.7% of all deaths globally.^[3]^ In US, stroke is the fifth most common reason for death, affecting nearly 7,95,000 individuals annually.^[4]^ The economic burden of stroke was calculated to be roughly 53 billion dollars from 2017 to 2018.^[5]^ With the social and economic burden of stroke continuing to increase, effective preventive measures and treatment strategies needed to be identified in order to reduce mortality and improve the quality of life of stroke patients.

Among the risk factors for the prognosis of stroke patients, cardiovascular health (CVH) has consistently been a significant concern. The primary risk factors for cardiovascular and cerebrovascular diseases include tobacco use, alcohol consumption, hypertension, diabetes, dyslipidemia, and atrial fibrillation.^[6]^ There was a substantial body of evidence indicated that maintaining optimal CVH could help reduce the risk of stroke.^[7]^ In 2010, the American Stroke Association launched a health initiative called Life’s Simple 7, which was used to evaluate an individual’s probability of experiencing cardiovascular disease (CVD).^[8]^ After 10 years, the assessment of sleep health was incorporated into Life’s Simple 7, which was defined as Life Essentials 8 (LE8). This new addition focused primarily on an individual’s lifestyle habits and physiological indicators.^[9]^ Recently, the significance of mental health has been increasingly recognized, a mental health assessment has been introduced in the context of LE8, defined as Life’s Crucial 9 (LC9). This assessment encompassed a range of factors, including dietary quality, physical activity, tobacco exposure, sleep health, body mass index, blood lipids, blood glucose, blood pressure, and mental health.^[10]^ Mental and physical health are inextricably intertwined, depression has emerged as a new independent risk factor for CVD.^[11]^ There was a positive association between depression and population-wide all-cause death.^[12]^

Although some studies have shown that LE8 scores were negatively correlated with cardiovascular mortality in stroke populations, the predictive value of LC9 in stroke populations needs to be further verified. This study analyzed clinical data from patients in the National Health and Nutrition Examination Survey (NHANES) database to explore the relationship between LC9 and all-cause and cardiovascular mortality in stroke populations, providing new methods and ideas for stroke prevention.

## 2. Materials and methods

The NHANES is a survey project initiated by the Disease Control and Prevention and the National Center for Health Statistics. It aims to understand the lifestyle and health trends of the American people through health and nutrition assessments. NHANES used stratified multistage sampling to select samples from different regions. Stratification was based on population size, ethnicity, and population distribution in order to guarantee that random samples were representative of entire US population. Data collection is mainly conducted through face-to-face interviews or telephone interviews, covering physical examinations, measurement of physiological indicators, surveys of eating habits, and socioeconomic information. Trained interviewers ensure the accuracy and reliability of the data by following a standardized process. The NHANES project began in 1996 and was published every 2 years, it can be found on the website (https://www.cdc.gov/nchs/nhanes/about/index.html). It provided researchers with the opportunity to analyze poor health issues and disease-related risk factors in American public, including, but not limited to unhealthy lifestyle choices, irregular eating patterns, and environmental influences. More research on NHANES has been mentioned in previous articles.^[13]^

### 2.1. Study population

**Figure 1.**
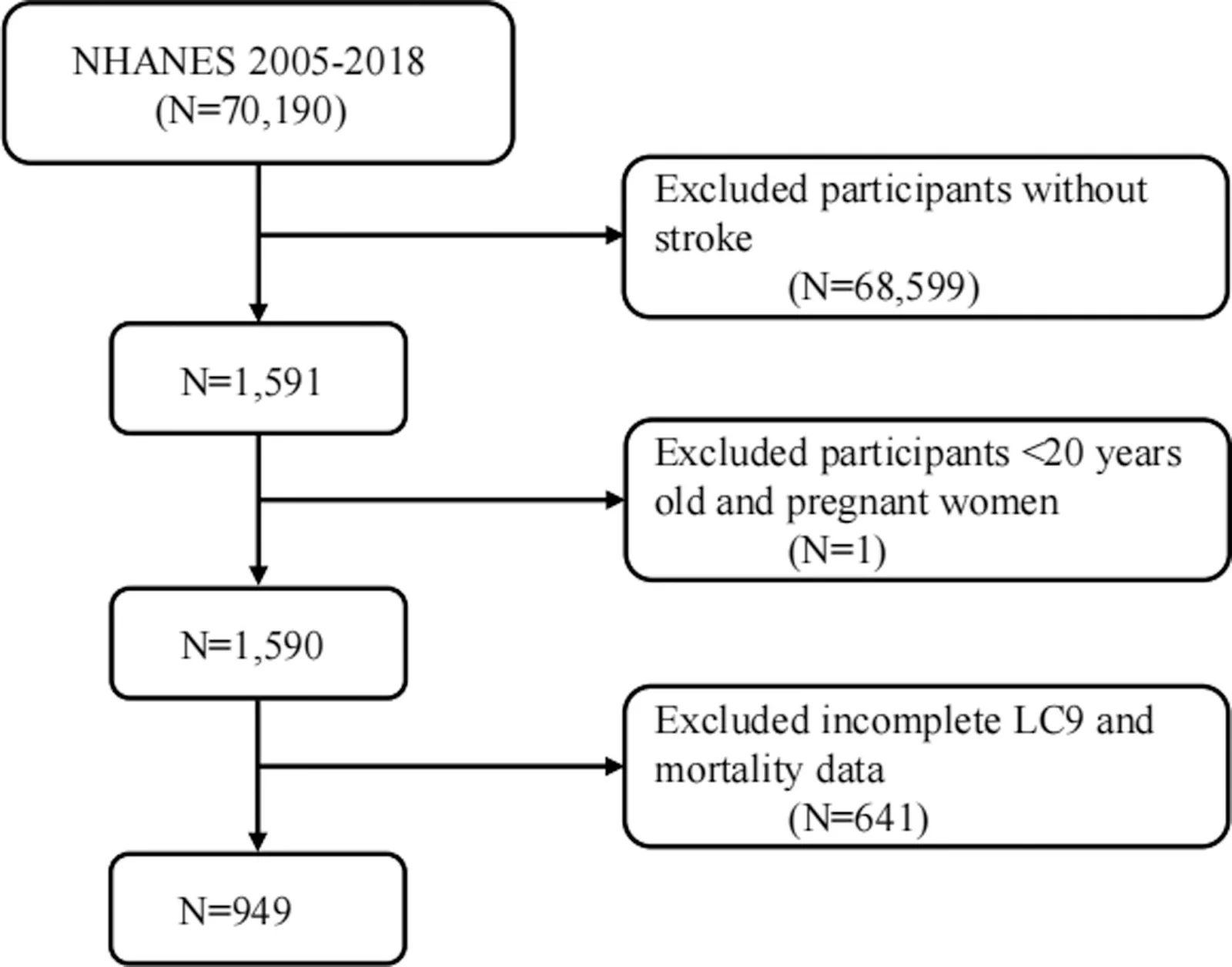
A flow diagram of eligible participant selection in the National Health and Nutrition. LC9 = Life’s Crucial 9, NHANES = National Health and Nutrition Examination Survey.

The present study drew upon data obtained from 70,190 participants between the years 2005 and 2018, of whom 1591 had been diagnosed with a stroke. After excluding subjects under the age of 20 pregnant women, and those with missing survival information and LC9 data, the study population was reduced to 949 individuals (Fig. 1).

### 2.2. Measurement of LC9

The LC9 score is a comprehensive tool based on the LE8 score and incorporates mental health assessment, which is used to comprehensively assess an individual’s CVH and mental health status. The LE8 score is composed of 8 key indicators: 4 health behaviors (diet, physical activity, smoking, and sleep) and 4 health factors (body mass index, non-high-density lipoprotein cholesterol, blood glucose, and blood pressure). Scoring is conducted for each indicator on a scale of 0 to 100, and the specific calculation method has been previously outlined in previous studies.^[14]^ LE8 score is calculated by combining 8 health indicators, a higher score indicates the better cardio-cerebral health. On this basis, the LC9 score is a comprehensive evaluation tool that incorporates a mental health assessment, utilizing the Patient Health Questionnaire-9 (PHQ-9) to evaluate depressive symptoms. A higher PHQ-9 score is representative of more intense depressive manifestations. The PHQ-9 score is categorized into 5 distinct categories: 0 to 4 points indicate no depression, 5 to 9 points indicate mild depression, and 10 to 14 points indicate moderate depression, corresponding to depression scores of 0, 25, 50, 75, and 100 points respectively.^[15]^ The LC9 score is calculated as the mean of the 8 indicators described above and the depression score.

### 2.3. Variables selection

Demographic and health factors were widely collected from the NHANES study (age, sex, race, marital status, education level, poverty-to-income ratio, diabetes, hypertension, and hyperlipidemia). The definition of specific data can be consulted in Table S1, Supplemental Digital Content, https://links.lww.com/MD/Q61.

### 2.4. Mortality results and follow-up time

This study used mortality data provided by the National Center for Health Statistics to obtain information on the study population’s deaths. The study’s outcomes encompassed all-cause mortality and mortality caused by CVD. All-cause mortality was death from any cause, and mortality caused by heart disease and cerebrovascular disease (international code ICD) was cardiovascular mortality. Data collection ended December 31, 2019 and the median follow-up time was 69 months. If a participant died during the follow-up period, the follow-up time was the interval between the interview date and the actual date of death. If the participant did not die, the follow-up period extended from the interview date until December 31, 2019.

### 2.5. Statistical analysis

Given the intricate multistage probability sampling methodology employed by the NHANES database, this study utilized WTDRD2 as the sample weights during the data processing stage. This approach was adopted to ensure the sample was nationally representative and to obtain analyses that more reliably reflected the actual characteristics of the overall US population. The WTDRD2 weights were derived by weighting the data from participants who completed the second 24-hour dietary recall interview, further enhancing the validity of the data.

In this study, continuous variables were expressed as the mean ± standard deviation, categorical variables were presented as counts (percentage). The differences between groups of continuous variables were compared using weighted *t*-tests, and the differences between categorical variables were compared using weighted chi-square tests. Cox proportional hazards regression models were used to explore the association between LC9 and all-cause and CVD mortality, with hazard ratio (HR) and 95% confidence interval (CI) calculated. In the regression analyses, model 1 was unadjusted, model 2 adjusted for confounders including age, sex, education level, marital status, poverty income ratio (PIR), and race, model 3 further adjusted for hypertension, diabetes, and hyperlipidemia on the basis of model 2.

The dose-response relationship between LC9 and mortality was analyzed using restricted cubic spline (RCS) modeling. Subgroup analyses were conducted based on specific baseline characteristics (age, sex, education level, marital status, PIR, education, hypertension, and diabetes) to identify potential interactions between LC9 and different stratification elements. Statistical significance was assigned a bilateral *P* < .05.

## 3. Results

### 3.1. Characteristics of the participants

Table 1 presented the baseline characteristics of the 949 patients included in the study, weighted to represent 34,81,831 US adults, with 24,15,033 (69%) weighted survivors and 10,66,798 (31%) deaths. Compared to the survivor group, the non-survivor group was older, had a heightened proportion of non-Hispanic White population, and showed a greater incidence of hypertension and obesity. Additionally, the non-survivor group had higher dietary scores and physical activity scores and significantly higher CVD mortality. Further details were provided in Table 1.

**Table 1:** The demographic characteristics of the stroke population in the present study were stratified by survival status, weighted.

Characteristic	Overall, N = 34,81,831 (100%)	Survivor, N = 24,15,033 (69%)	Non-survivors, N = 10,66,798 (31%)	*P*-Value
Number of participants in the sample	949	636	313	**–**
Age (%)				**<.001**
* *20–65	16,36,718 (47%)	14,22,470 (59%)	2,14,248 (20%)	
* >*65	18,45,114 (53%)	9,92,563 (41%)	8,52,550 (80%)	
Sex (%)				.33
* *Female	20,12,339 (58%)	14,29,139 (59%)	5,83,201 (55%)	
* *Male	14,69,492 (42%)	9,85,894 (41%)	4,83,598 (45%)	
Race (%)				**<.001**
* *Non-Hispanic White	25,75,015 (74%)	16,64,373 (69%)	9,10,642 (85%)	
* *Non-Hispanic Black	4,78,426 (14%)	3,83,713 (16%)	94,713 (8.9%)	
* *Other	2,83,215 (8.1%)	2,51,996 (10%)	31,219 (2.9%)	
* *Mexican American	1,45,175 (4.2%)	1,14,951 (4.8%)	30,224 (2.8%)	
Married/live with partner (%)				**.044**
* *no	14,01,734 (40%)	9,03,232 (37%)	4,98,502 (47%)	
* *yes	20,80,097 (60%)	15,11,801 (63%)	5,68,296 (53%)	
Education level (%)				.11
* *Below high school	8,63,300 (25%)	5,55,678 (23%)	3,07,622 (29%)	
* *High school or above	26,18,531 (75%)	18,59,355 (77%)	7,59,176 (71%)	
PIR (%)				.55
* *Poor	9,89,699 (30%)	7,08,823 (31%)	2,80,876 (28%)	
* *Not poor	2266,658 (70%)	15,61,638 (69%)	7,05,020 (72%)	
Hypertension (%)				**.007**
* *no	7,18,692 (21%)	5,62,418 (23%)	1,56,274 (15%)	
* *yes	27,63,140 (79%)	18,52,615 (77%)	9,10,525 (85%)	
Diabetes (%)				.14
* *no	22,11,964 (64%)	15,81,029 (65%)	6,30,935 (59%)	
* *yes	12,69,867 (36%)	8,34,004 (35%)	4,35,864 (41%)	
Hyperlipidemia (%)				.60
* *no	4,23,432 (12%)	2,83,553 (12%)	1,39,879 (13%)	
* *yes	30,58,399 (88%)	21,31,480 (88%)	9,26,919 (87%)	
Mean LC9 score (mean (SD))	59.15 (14.65)	59.54 (14.90)	58.26 (14.05)	.18
Mean psychological health score (mean (SD))	76.79 (33.39)	74.89 (35.17)	81.09 (28.54)	.075
Mean HEI-2015 diet score (mean (SD))	35.45 (30.07)	32.21 (29.62)	42.77 (29.84)	**<.001**
Mean physical activity score (mean (SD))	48.35 (47.27)	55.72 (47.09)	31.68 (43.34)	**<.001**
Mean tobacco exposure score (mean (SD))	64.55 (39.41)	63.30 (40.83)	67.38 (35.88)	.93
Mean sleep health score (mean (SD))	76.04 (29.96)	74.82 (30.64)	78.82 (28.23)	.15
Mean body mass index score (mean (SD))	53.19 (34.12)	51.13 (34.24)	57.85 (33.44)	**.021**
Mean blood lipid score (mean (SD))	60.88 (29.39)	61.81 (28.61)	58.80 (31.02)	.34
Mean blood glucose score (mean (SD))	68.50 (30.50)	70.26 (30.58)	64.52 (29.99)	**.025**
Mean blood pressure score (mean (SD))	48.59 (32.37)	51.74 (32.11)	41.46 (31.86)	**.001**
LC9, tertile (%)				.18
* *T1	11,26,370 (32%)	7,60,606 (31%)	3,65,764 (34%)	
* *T2	11,80,044 (34%)	7,88,140 (33%)	3,91,904 (37%)	
* *T3	11,75,417 (34%)	8,66,287 (36%)	3,09,130 (29%)	
Cardiovascular mortality (%)	2,93,380 (8.4%)	0 (0%)	2,93,380 (28%)	**<.001**

T1–T3: Grouped by tertile according to LC9. Mean (SD) for continuous variables: the *P*-value was calculated by the weighted t test. Percentages (weighted N, %) for categorical variables: the *P*-value was calculated by the weighted chi-square test. Statistically significant values (*P* < .05) are indicated in bold. LC9 = Life’s Crucial 9, PIR = poverty income ratio, SD = standard deviation, T1–T3 = tertiles 1–3.

### 3.2. Association of LC9 with all-cause and cardiovascular mortality in stroke population

The association between LC9 and mortality was analyzed using 3 adjusted Cox proportional hazards models. Table 2 demonstrated that higher LC9 was associated with reduced all-cause and cardiovascular mortality. When LC9 was treated as a continuous variable (per 10-point increase), the risk of all-cause mortality significantly decreased (HR = 0.83, 95% CI: 0.74–0.94). When LC9 was categorized into tertiles, participants in the highest tertile exhibited a 48% reduced risk of all-cause mortality compared to those in the lowest tertile (HR = 0.52, 95% CI: 0.35–0.78), which was consistent with the results observed in model 1 and model 2.

**Table 2:** HRs (95% CIs) for all-cause mortality and cardiovascular mortality according to LC9 in the stroke population.

LC9	Model 1 [HR (95% CI)]	*P*-value	Model 2 [HR (95% CI)]	*P*-value	Model 3 [HR (95% CI)]	*P*-value
All-cause mortality						
Continuous (per 10 scores)	0.83 (0.75,0.91)	<.001	0.86 (0.77,0.95)	.004	0.83 (0.74,0.94)	.003
Tertile						
T1	1 (ref.)		1 (ref.)		1 (ref.)	
T2	0.90 (0.63,1.30)	.900	0.93 (0.65,1.33)	.670	0.89 (0.62,1.28)	.530
T3	0.49 (0.35,0.69)	<.001	0.56 (0.40,0.80)	.001	0.52 (0.35,0.78)	.001
* P* for trend	<.001		.001		.001	
Cardiovascular mortality						
Continuous (per 10 scores)	0.73 (0.61, 0.88)	<.001	0.76 (0.61, 0.93)	.010	0.71 (0.57, 0.89)	.003
Tertile						
T1	1 (ref.)		1 (ref.)		1 (ref.)	
T2	0.73 (0.40, 1.31)	.290	0.75 (0.42, 1.33)	.320	0.63 (0.35, 1.12)	.110
T3	0.29 (0.13, 0.61)	<.001	0.31 (0.14, 0.68)	.004	0.26 (0.12, 0.56)	<.001
* P* for trend	<.001		.002		<.001	

T1–T3: Grouped by tertile according to LC9.

Model 1: No covariates were adjusted.

Model 2: Age, sex, education level, marital status, PIR, and race were adjusted.

Model 3: Age, sex, education level, marital status, PIR, race, hypertension, diabetes, and hyperlipidemia were adjusted.

CI = confidence interval, HR = hazard ratio, LC9 = Life’s Crucial 9, PIR = poverty income ratio, T1–T3 = tertiles 1–3.

**Figure 2.**
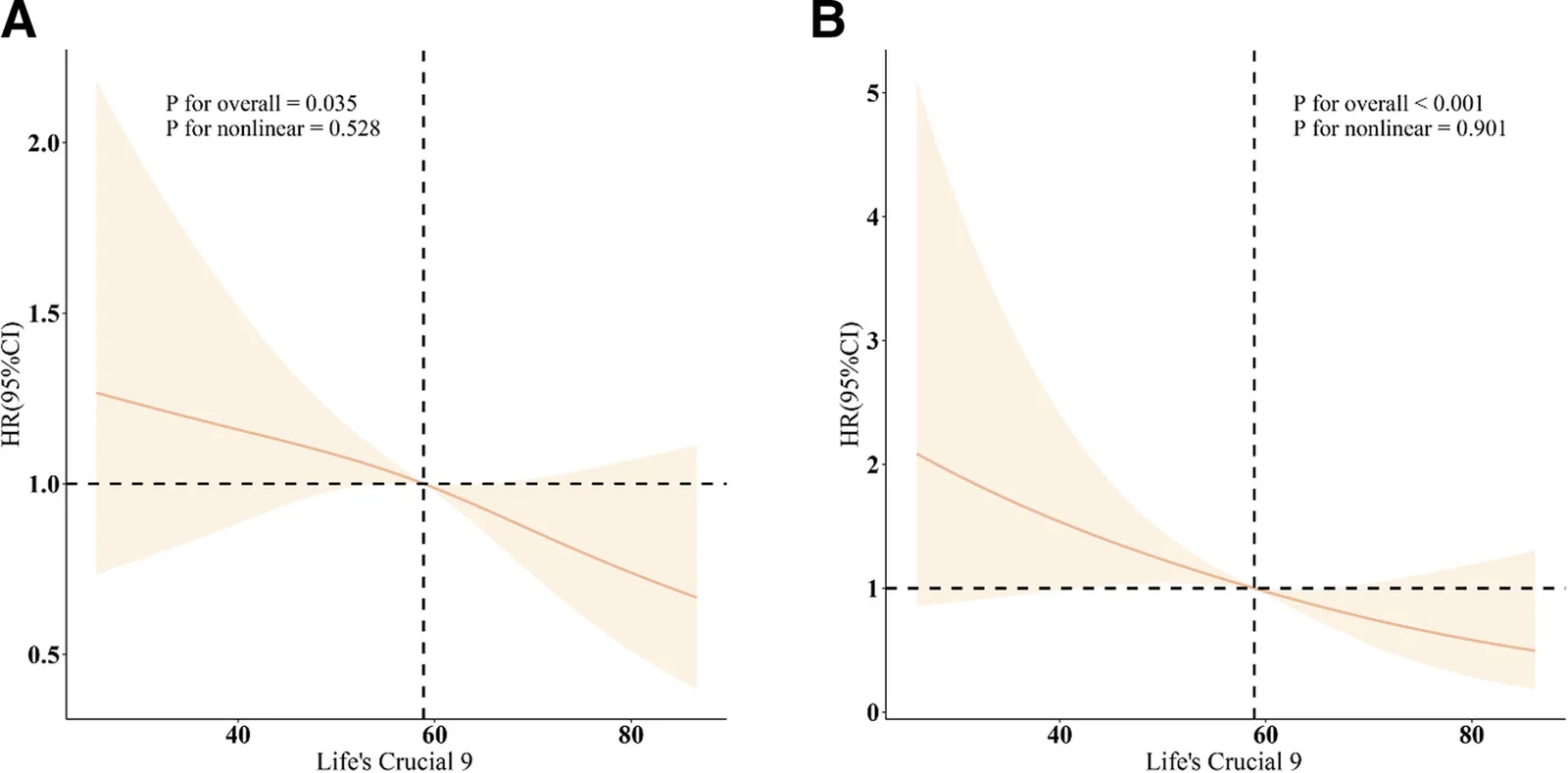
The association of LC9 with All-cause (A) and cardiovascular mortality (B) among stroke population visualized by restricted cubic spline. HR (solid lines) and 95% CI (shaded areas) were adjusted for age, sex, education level, marital status, PIR, race, hypertension, diabetes, and hyperlipidemia. CI = confidence interval, HR = hazard ratio, LC9 = Life’s Crucial 9, PIR = poverty income ratio.

**Figure 3.**
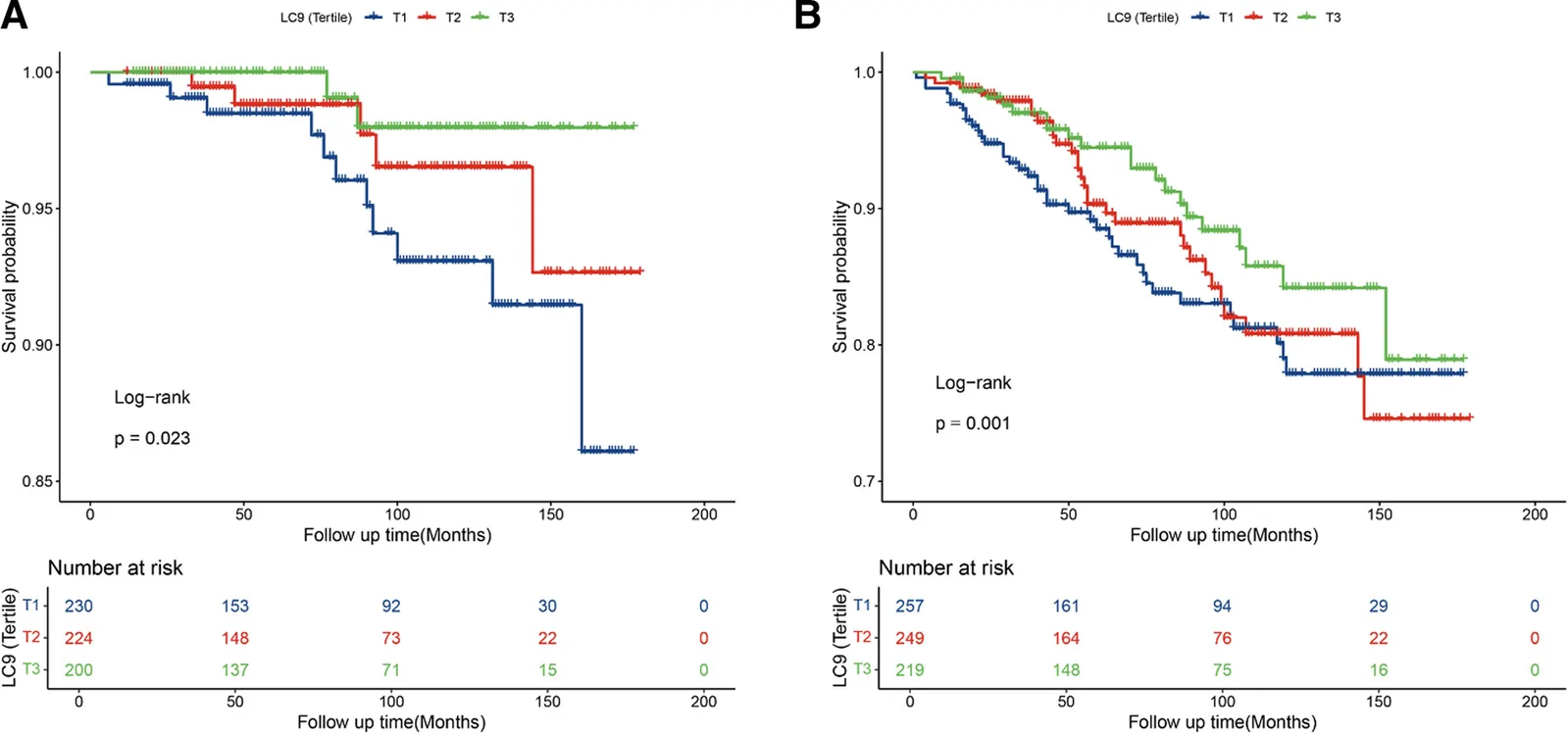
Kaplan–Meier analyses for mortality among the 3 groups. (A) All-cause mortality; (B) cardiovascular mortality. LC9 = Life’s Crucial 9, T1–T3 = tertiles 1–3.

Similarly, a consistent trend was also identified in cardiovascular mortality. When LC9 was analyzed as a continuous variable, a higher LC9 was significantly associated with a reduced risk of cardiovascular mortality (HR = 0.71, 95% CI: 0.57–0.89). In the unadjusted model, participants in the highest tertile of LC9 exhibited an 81% lower risk of cardiovascular death (HR = 0.29, 95% CI: 0.13–0.61) compared to those in the lowest tertile. After adjusting for potential confounders, this association remained robust, with the highest tertile group showing a significantly reduced risk of cardiovascular mortality (HR = 0.26, 95% CI: 0.12–0.56). These findings suggested that higher LC9 scores were independently associated with a lower risk of cardiovascular death. Additionally, RCS analysis revealed a linear association between LC9 scores and both all-cause and cardiovascular mortality (Fig. 2). The Kaplan–Meier analysis demonstrated that patients in the LC9 groups with higher scores exhibited significantly higher survival rates, both in terms of all-cause mortality and cardiovascular mortality, and that this advantage persisted as the follow-up time increased (Fig. 3).

### 3.3. Subgroup analysis

**Figure 4.**
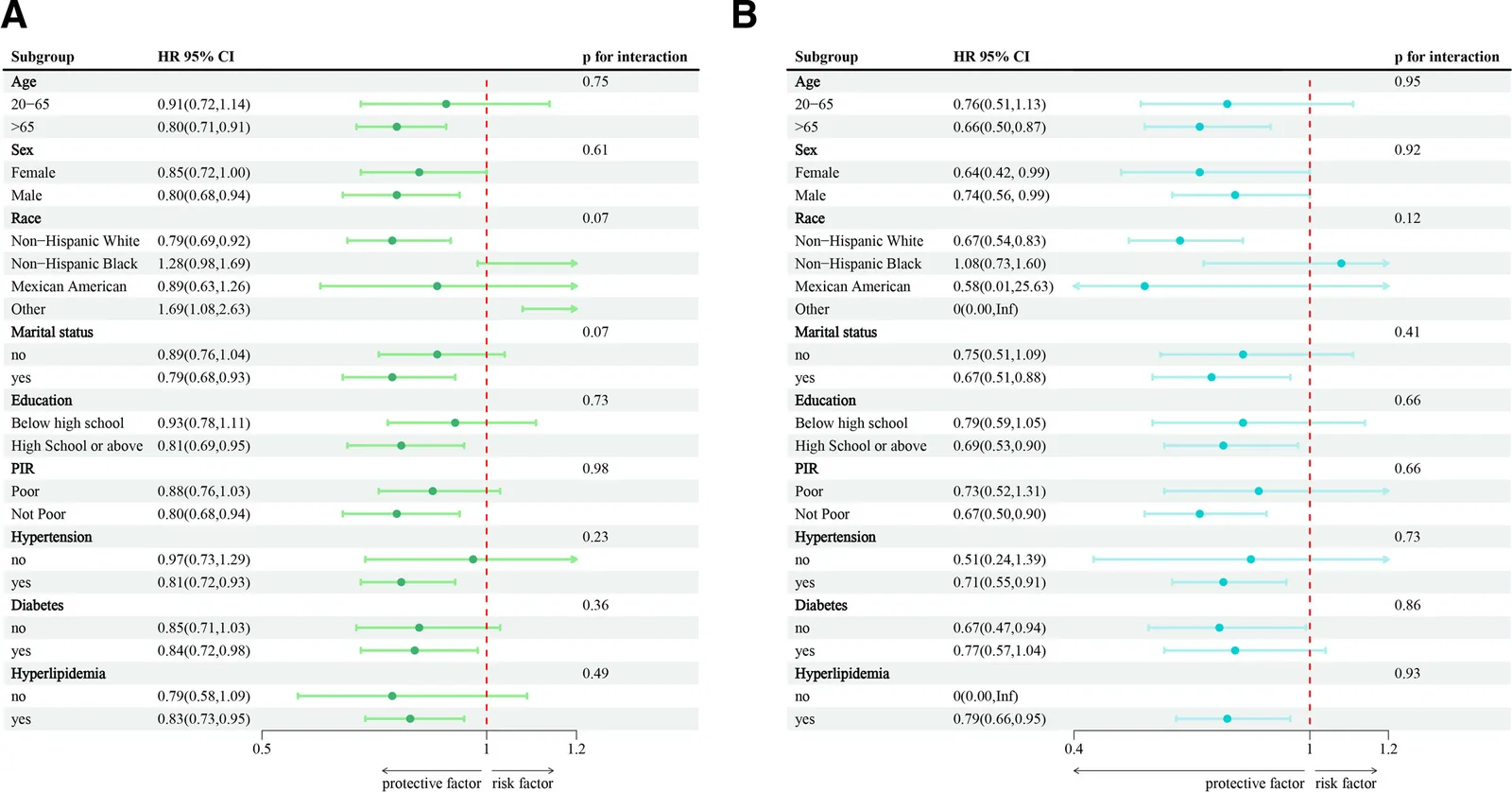
Subgroup analysis between LC9 and mortality. (A) LC9 – all-cause mortality; (B) LC9 – cardiovascular mortality. Analyses were adjusted for age, sex, education level, marital status, PIR, race, hypertension, diabetes, and hyperlipidemia. CI = confidence interval, HR = HR = hazard ratio, LC9 = Life’s Crucial 9, PIR = poverty income ratio.

In subgroup analyses, the association between LC9 and mortalities was consistent across various subgroups (Fig. 4). No substantial interactions were observed with respect to age, sex, marital status, education level, hypertension, poverty status, diabetes, or dyslipidemia (all interaction *P*-values > .05). These results suggested that the predictive value of LC9 for mortality outcomes was robust and not significantly influenced by these demographic or clinical factors.

## 4. Discussion

This study examined the association between LC9 and mortality in stroke patients, specifically investigating the relationship between the score and both cardiovascular mortality and all-cause mortality. Higher LC9 were associated with improved long-term survival and reduced risks of both all-cause mortality (HR = 0.52, 95% CI: 0.35–0.78) and cardiovascular mortality (HR = 0.29, 95% CI: 0.13–0.61). RCS analyses revealed a linear inverse relationship between LC9 and mortality risks, while survival curves further confirmed that patients with higher LC9 values experienced significantly better outcomes and a reduced risk of mortality. This suggested that adherence to ideal CVH behaviors, combined with favorable mental health status, may synergistically mitigate poststroke mortality.

Compared to the previous LE8, our study utilized LC9 as a new composite score that integrated LE8 and depression severity. Based on existing evidence that LE8 could protect CVH,^[16]^ we also observed that LC9 was inversely related to poststroke mortality after incorporating mental health factors. Previous studies have emphasized the importance of mental health for CVH. A meta-analysis comprising 28 studies demonstrated that depression was positively correlated with both stroke occurrence and mortality in stroke patients.^[17]^ Furthermore, a prospective study of Chinese adults demonstrated that depression was associated with an enhanced risk of all-cause mortality and cardiovascular mortality, with this association being particularly evident in male subjects.^[18]^

At present, the relationship between depression and CVD has attracted widespread attention in the field of public health. A plethora of studies had demonstrated that depression not only increases the risk of all-cause mortality and cardiovascular mortality in patients but was also associated with cognitive impairment, dementia, and delayed recovery after stroke.^[19–21]^ As an independent risk factor for stroke,^[22]^ depression could interact synergistically with traditional risk factors such as hypertension and hyperlipidemia,^[23]^ exacerbating the health burden on individuals by affecting the nervous system.^[24]^ Therefore, incorporating mental health into the comprehensive assessment of CVH holds preventive importance.

The new LC9 indicators encompassed smoking, non-high-density lipoprotein cholesterol, blood pressure, body mass index, blood glucose, physical activity, diet quality, sleep duration, and mental health. A large cohort study of individuals aged 70 years demonstrated that physical activity was associated with a reduced risk of stroke, myocardial infarction, and all-cause mortality. The protective effect of moderate-intensity physical activity was particularly pronounced and effectively mitigated the adverse effects of sedentary behavior.^[25]^ Meanwhile, physical activity could reduce the risk of death in patients diagnosed with CVD.^[26]^ In addition, sleep deprivation and an unhealthy diet are closely linked to the occurrence of cardiovascular events. According to another meta-analysis found that in healthy people, the risk of CVD increases by 6% for every hour of sleep lost.^[27]^ Large prospective cohort studies have shown that a healthy diet, including the Mediterranean diet, helps reduce the risk of CVD and death.^[28,29]^

The protective effect of LC9 may be driven jointly by CVH behaviors and mental health, which help individuals directly identify and improve risk factors to improve their scores. The precise mechanism underlying the association between LC9 and poor prognosis in stroke patients remains unclear, however, previous studies have proposed that it may be linked to the following potential mechanisms. Inadequate management of blood pressure has been demonstrated to engender a number of deleterious effects, including the diminution of oxidative stress and nitric oxide release, the attenuation of vascular endothelial dilation, and the acceleration of the progression of atherosclerosis.^[30,31]^ The process of arterial stiffening has been shown to result in a decrease in the sensitivity of the carotid baroreceptors, triggering excessive activation of the sympathetic nervous system, which in turn places an additional burden on the cardiovascular system.^[32–34]^ Furthermore, in patients diagnosed with depression, excessive stress has been demonstrated to induce a disruption in neuroendocrine regulation, thereby precipitating heightened activity within the sympathetic nervous system and leading to overactivation of the hypothalamic-pituitary-adrenal axis. The former promotes the release of adrenaline, while the latter leads to increased secretion of cortisol, which elevates myocardial oxygen consumption and increases blood pressure, thereby increasing the risk of cardiovascular events.^[35,36]^ Secondly, depression releases elevated levels of inflammatory factors (such as C-reactive protein and pro-inflammatory cytokines), damages blood vessel cells, and triggers atherosclerosis and adverse cardiovascular events.^[37–40]^ Finally, depression lowers patients’ compliance with drug therapy and rehabilitation training and may lead to the maintenance of risky behaviors such as smoking and alcohol abuse, which reduce the LC9 score and speed up vascular disease indirectly.^[41]^

This study’s strength relies on the application of substantial sample size derived from NHANES database, and the first inclusion of depression assessment in the LC9 score, which provides a new perspective on the assessment of mortality risk in the stroke population. However, some limitations should be acknowledged. Lifestyle factors (dietary habits, sleep duration, and mental health status) were self-reported and may introduce recall bias. Second, as a cohort study, causal inference cannot be directly inferred, and future randomized trials are needed to test whether LC9-guided interventions can directly reduce mortality. Third, this study includes cardiovascular deaths caused by heart disease or cerebrovascular disease. Future studies can analyze and discuss the mortality outcomes caused by each separately to reduce potential bias. Finally, the stroke patients were from the US, and owing to the potential impact of racial and regional differences, that may limit the global applicability of the research.

## 5. Conclusions

This study demonstrated that an augmentation in the LC9 was evidently associated with a diminution in all-cause mortality and cardiovascular mortality in stroke patients. The incorporation of depression assessment into routine cardiovascular risk indicators elucidated the common role of mental health and CVH in prognosis. It provided a novel perspective for the prognosis management of stroke patients.

## Acknowledgments

We are very grateful to the NHANES study participants for their contribution.

## Author contributions

**Data curation:** Wanhong Zhang, Jiaying Li.

**Formal analysis:** Wanhong Zhang.

**Methodology:** Jiaying Li.

**Validation:** Wenbo Zhang, Henghao Wu.

**Writing – original draft:** Wenbo Zhang, Henghao Wu, Xinsheng Han.

**Writing – review & editing:** Wanhong Zhang, Xinsheng Han.

## Supplementary Material
